# The stressosome is required to transduce low pH signals leading to increased transcription of the amino acid-based acid tolerance mechanisms in *Listeria monocytogenes*

**DOI:** 10.1099/acmi.0.000455

**Published:** 2022-09-14

**Authors:** Duarte N. Guerreiro, Aoife Boyd, Conor P. O'Byrne

**Affiliations:** ^1^​ Bacterial Stress Response Group, Microbiology, School of Biological and Chemical Sciences, National University of Ireland, Galway, Ireland; ^2^​ Pathogenic Mechanisms Research Group, Microbiology, School of Natural Sciences, National University of Ireland, Galway, Ireland

**Keywords:** acid adaptation, general stress response, kinase RsbT, *Listeria monocytogenes*, RsbR1, sigB, stressosome

## Abstract

Increasing proton concentration in the environment represents a potentially lethal stress for single-celled microorganisms. To survive in an acidifying environment, the foodborne pathogen *Listeria monocytogenes* quickly activates the alternative sigma factor B (σ^B^), resulting in upregulation of the general stress response (GSR) regulon. Activation of σ^B^ is regulated by the stressosome, a multi-protein sensory complex involved in stress detection and signal transduction. In this study, we used *L. monocytogenes* strains harbouring two stressosome mutants to investigate the role of this complex in triggering expression of known amino acid-based resistance mechanisms in response to low pH. We found that expression of glutamate decarboxylase (*gadD3*) and arginine and agmatine deiminases (*arcA* and *aguA1*, respectively) were upregulated upon acid shock (pH 5 for 15 min) in a stressosome-dependent manner. In contrast, transcription of the *arg* operons (*argGH* and *argCJBDF*), which encode enzymes for the l-arginine biosynthesis pathway, were upregulated upon acid shock in a stressosome-independent manner. Finally, we found that transcription of *argR*, which encodes a transcriptional regulator of the *arc* and *arg* operons, was largely unaffected by acidic shock. Thus, our findings suggest that the stressosome plays a role in activating amino acid-based pH homeostatic mechanisms in *L. monocytogenes *. Additionally, we show that genes encoding the l-arginine biosynthesis pathway are highly upregulated under acidic conditions, suggesting that intracellular arginine can help withstand environmental acidification in this pathogen.

## Introduction

The foodborne pathogen *Listeria monocytogenes *, the aetiological agent of listeriosis, is a robust bacterium capable of surviving in harsh environments including the extremely low pH of the human stomach [1, 2]. *L. monocytogenes* senses acidification of the environment through a multi-protein complex designated as the stressosome, composed of putative sensory proteins RsbR1 and its four paralogues, the scaffold protein RsbS and the serine–threonine kinase RsbT, which is responsible for the phosphorylation of RsbR1 and RsbS [3, 4]. Under stressful conditions, the stressosome activates a signal cascade that ultimately releases the alternative sigma factor B (σ^B^) from an anti-sigma factor, culminating in the upregulation of approximately 300 genes that comprise the general stress response (GSR) regulon. A range of homeostatic and protective mechanisms are encoded by the GSR regulon that are responsible for enhancing resistance to lethal stresses, including extreme acidic conditions [5–8]. The σ^B^ regulon also encompasses some virulence factors of *L. monocytogenes,* such as the internalins *inlA* and *inlB* [9–12], and mutants lacking σ^B^ (Δ*sigB*) exhibit attenuated virulence in intragastrically inoculated guinea pigs [12, 13].

**Fig. 1.**
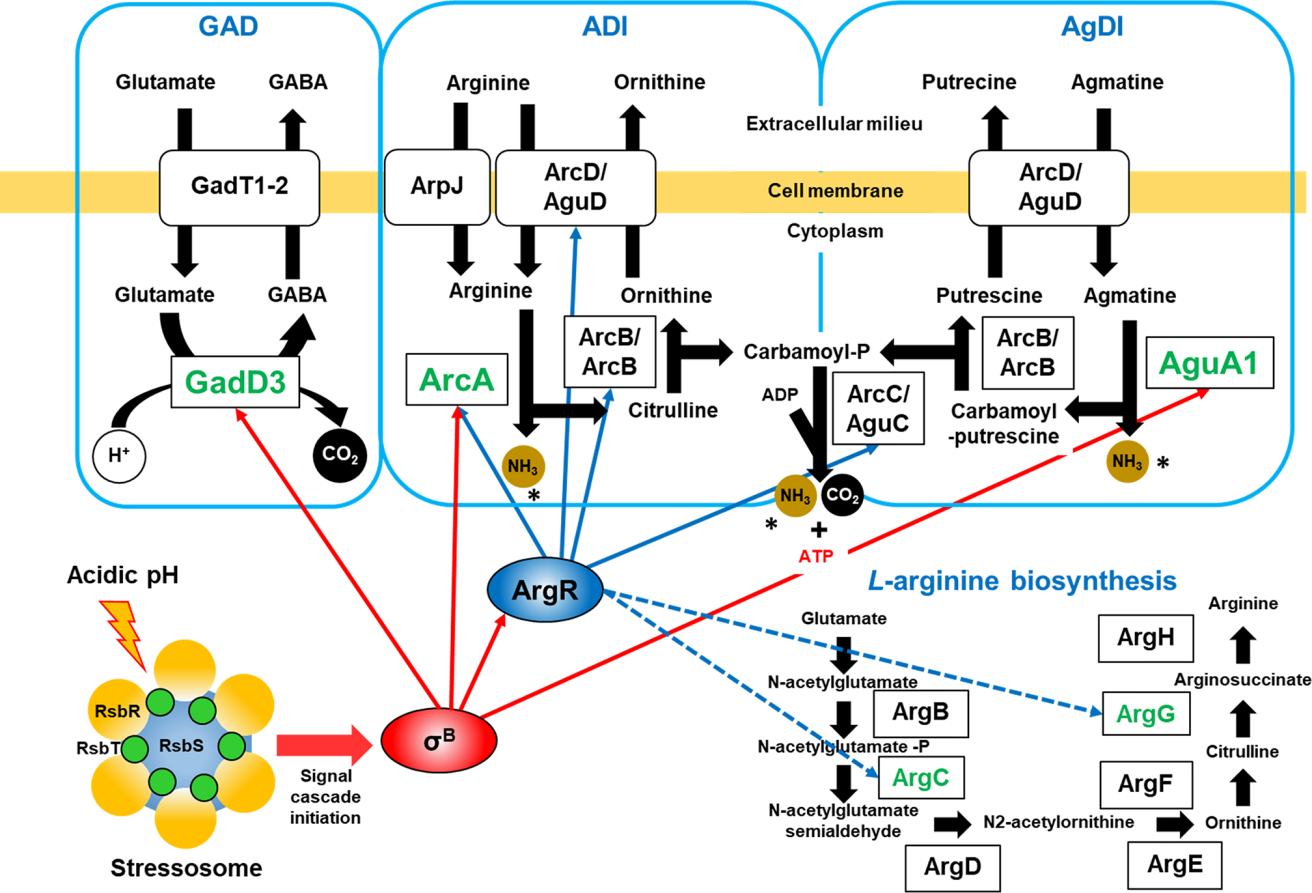
σ^B^ and ArgR regulatory network over the GAD, ADI and AgDI systems. Schematic representation of the regulatory functions of the stressosome, σ^B^ and the transcriptional regulator ArgR, the metabolic pathways glutamate decarboxylase, arginine and agmatine deiminase systems and l-arginine biosynthesis pathway in *L. monocytogenes *. The enzymes depicted in green (GadD3, ArcA and AguA1) correspond to the genes *gadD3*, *arcA* and *aguA1*, respectively, analysed in this study by RT-qPCR. Red and blue arrows represent gene upregulation while dashed blue arrows represent gene repression in stationary phase grown cells identified in other studies. *The newly formed ammonia (NH_3_) reacts with protons (H^+^) forming ammonium (NH_4_^+^), which neutralizes the cytosolic pH.

It is well known that *L. monocytogenes* can increase acid tolerance in response to sub-lethal acid exposure, a response known as the adaptive acid tolerance response (ATR) [14, 15]. σ^B^ probably contributes to this response since it is activated at the same low pH values that trigger the ATR, although some researchers have suggested that it is not the main regulator controlling the response [16]. In a recent study, we demonstrated the pivotal role of the stressosome in the sensing of low pH and the subsequent activation of σ^B^ [3]. Pre-treating mid-log phase cultures at pH 5 for 15 min increased the transcription of highly σ^B^-dependent genes *lmo2230* and *lmo0596*, and enhanced *L. monocytogenes* acid tolerance in a stressosome-dependent manner. The genes *lmo2230* and *lmo0596* encode a putative arsenate reductase and a transmembrane protein with unknown function, respectively [3]. It is currently unknown whether the stressosome is required for upregulation of the amino acid-based acid resistance mechanisms in response to acidification of the medium, although σ^B^ is known to play a role in regulating some elements of this system, including the glutamate decarboxylase (GAD) system [17] and the arginine deiminase (ADI) system [18] (Fig. 1).

The *L. monocytogenes* GAD system plays a critical role in acid tolerance by consuming protons (H^+^) through the decarboxylation of l-glutamate into γ-aminobutyrate (GABA) [19–23]. This system comprises two glutamate/GABA antiporters, GadT1 (Lmo0448) and GadT2 (Lmo2362), and three glutamate decarboxylases, GadD1 (Lmo0448), GadD2 (Lmo2363) and GadD3 (Lmo2434), of which only GadD3 is known to be σ^B^-dependent [24]. Similarly, the ADI system contributes to *L. monocytogenes* acid tolerance by metabolizing l-arginine into citrulline and ammonia (NH_3_), which serves to buffer the cytoplasmic pH [21, 25, 26]. Citrulline is further metabolized to ornithine and carbamoyl-phosphate and the latter is subsequently metabolized to ATP, CO_2_ and NH_3_. The ADI system is also induced by σ^B^ and comprises the arginine/ornithine antiporter ArcD (Lmo0037, also known as AguD), catabolic ornithine carbamoyltransferase ArcB (Lmo0036, also known as AguB), the carbamate kinase ArcC (Lmo0039, also known as AguC) and the arginine deiminase ArcA (Lmo0043) [18]. An additional acid tolerance mechanism, the agmatine deiminase (AgDI) system, was identified in *L. monocytogenes* and like the GAD and ADI systems, AgDI plays a role in acid tolerance [21, 25, 27–30] and is upregulated at pH 5 [18, 28]. Except for the agmatine deiminase AguA1 (Lmo0038), which metabolizes agmatine into carbamoyl-putrescine, the components of ADI are shared with AgDI, known as either *arc* or *agu* genes [29]. While the stressosome is known to transduce acid signals [3], thus far its role in regulating these amino acid-based resistance mechanisms has not been studied.

In addition to σ^B^, the ADI system is regulated by the transcriptional regulator ArgR [18, 29]. ArgR (homologous to AhrC in *Bacillus subtilis *) consists of a DNA binding transcriptional activator of the ADI system and a repressor of l-arginine biosynthesis in several bacterial species [31–36]. This regulator is implicated in the acid tolerance of *L. monocytogenes* [18, 29]. In the presence of l-arginine, ArgR suppresses the transcription of two operons encoding the arginine biosynthesis pathway, the *argCJBDF* operon (*lmo1591–lmo1587*, respectively) and *argGH* (*lmo2090* and *lmo2091*, respectively) [18, 29]. Interestingly, Cheng and colleagues demonstrated that ArgR also binds to the promoter region of *rsbV* at the *rsbVW-sigB-rsbX* operon and suppresses the transcription of *sigB* in the absence of arginine [29]. However, this regulatory effect is likely to be minor, with the partner-switching pathway regulated by the stressosome playing the dominant role in controlling activity of σ^B^. ArgR is implicated in acid tolerance as an Δ*argR* strain exhibits increased acid tolerance 90 min after the onset of stress (pH 3.5) [29]. In addition, *argR* is upregulated by σ^B^ in cultures grown to stationary phase [18]. To our knowledge, it is currently unknown whether σ^B^ influences the transcription of *argR* under conditions of mild acid stress.

In this study, we aimed to extend current knowledge of the role of the stressosome in regulating expression of the acid tolerance mechanisms such as GAD, ADI and AgDI in *L. monocytogenes *. Here, we analysed the transcriptional response to acidification of the medium of the genes *gadD3, arcA* and *aguA1*, integral components of the GAD, ADI and AgDI systems, as well as the *argR*, *argC* and *argG* genes. We found that a σ^B^ knockout deletion strain (Δ*sigB*) and RsbT_N49A_, a stressosome inactive strain unable to phosphorylate RsbR1 and RsbS, were unable to upregulate *gadD3*, *arcA* or *aguA1*. Our data show a critical role for the stressosome in regulation of the amino acid-based pH homeostatic mechanisms employed by *L. monocytogenes* to withstand the detrimental effects of acidification of the environment.

## Methods

### Bacterial strains and primers

*L. monocytogenes* EGD-e (serovar 1/2 a), isogenic mutant strains and primers used in this study are listed in Table 1. Strains were grown in BHI broth (LabM) at 37 °C with constant shaking at 150 rpm at initial neutral pH of ~7.4.

**Table 1.** Strains and primers used in this study

Strain/mutant	Source
* Listeria monocytogenes* EGD-e	K. Boor
* L. monocytogenes* EGD-e Δ*sigB*	[47]
* L. monocytogenes* EGD-e RsbT_N49A_	[4]
* L. monocytogenes* EGD-e RsbL_C56A_; Δ*rsbR2*; Δ*rsbR3*; Δ*rsbR4*	[4]
**Primer (5′−3**′)	**Target**
TGGGGAGCAAACAGGATTAG	16S_F
TAAGGTTCTTCGCGTTGCTT	16S_R
GAAACGCTCGAGAAAAATGC	*gadD3*_F
AGTTTGGTCGTTTTGCCTGT	*gadD3*_R
GGTCGCAAATTAGAAGTGCATAA	*aguA1*_F
GGATCCCCAAATAGCGGAAAA	*aguA1*_R
GGCGGAGAAGATGTAATTGTTTC	*arcA*_F
CCCGCACTTCTTAACAGATCG	*arcA*_R
CCCACATCAAAAACTAAAACGCG	*argR*_F
GGCCAGTCCAAGTTATCGATTAA	*argR*_R
CCTTTGTTCGTGAAGTGGCA	*argG*_F
CCTTTAAATAATTTGACGCGGATGG	*argG*_R
CGCCCCTTTGACTAAATTATCAAT	*argC*_F
CCGAATCCAACCAGAGAATGTATA	*argC*_R

GAD, glutamate decarboxylase.

### Acid shock treatment in *L. monocytogenes*

*L. monocytogenes* strains were grown to stationary phase cultures at 37 °C for 16 h followed by dilution to an initial OD_600 nm_ of 0.05 in fresh BHI. Cultures were allowed to grow at 37 °C to the mid-log phase (OD_600 nm_ of 0.4). Acid shock-treated cultures were made by adding 5 M HCl until pH 5 was reached. Treated (+) and untreated (-) cultures were incubated for a further 15 min at 37 °C. Three independent biological replicates were made.

### RNA extraction and RT-qPCR

To stop transcription, cultures were diluted in RNAlater (Sigma) at a 1 : 5 ratio. The total RNA was extracted using an RNeasy Minikit (Qiagen) according to the manufacturer’s recommendations. Cells were disrupted by bead beating twice using the FastPrep-24 (MP Biomedicals) at a speed of 6 m s^−1^ for 40 s. DNA was digested with Turbo DNA-free (Invitrogen) according to the manufacturer’s recommendations. RNA integrity was verified by electrophoresis in 0.7 % (w/v) agarose gels. Synthesis of cDNA was performed with a SuperScript III First-Strand Synthesis System (Invitrogen) according to the manufacturer’s recommendations. cDNA was quantified using a NanoDrop 2000c (Thermo Scientific) and diluted to a final concentration of 7 ng ml^−1^. Real-time quantitative PCR (RT-qPCR) was performed using the QuantiTect SYBR Green PCR kit (Qiagen) and pair of primers for the target genes (Table 1). Primer efficiency for 16S, *gadD3*, *arcA*, *aguA1*, *argR*, *argG* and *argC* were previously determined using cDNA [3]. Samples were analysed on the LightCycler 480 system (Roche) with the following parameters: 95 °C for 15 min; 45 cycles of 15 s at 95 °C, 15 s at 53 °C and 30 s at 72 °C; a melting curve drawn for 5 s at 95 °C and 1 min at 55 °C, followed by increases of 0.11 °C s^−1^ until 95 °C was reached; and cooling for 30 s at 40 °C. Cycle quantification values were calculated by using LightCycler 480 software version 1.5.1 (Roche) and the Pfaffl relative expression formula [37, 38]. Expression of 16S rRNA was used as a reference gene. Expression of the 16S rRNA gene remained stable and unresponsive towards the acid shock treatment in all strains and biological replicates. Results are expressed as Log_2_ relative expression ratios normalized against average expression of the *L. monocytogenes* wild-type (WT) strain in the absence of stress.

### Statistical analysis

All statistical analyses were performed by conducting unpaired Student’s *t*-tests with GraphPad Prism 8. All analyses were made by comparing each strain with the untreated *L. monocytogenes* WT strain. *P* values of <0.05 (*), <0.01 (**) and <0.001 (***) were considered statistically significant.

## Results and discussion

### Expression of *gadD3*, *arcA* and *aguA1* is stressosome-dependent under mild acidic conditions

**Fig. 2.**
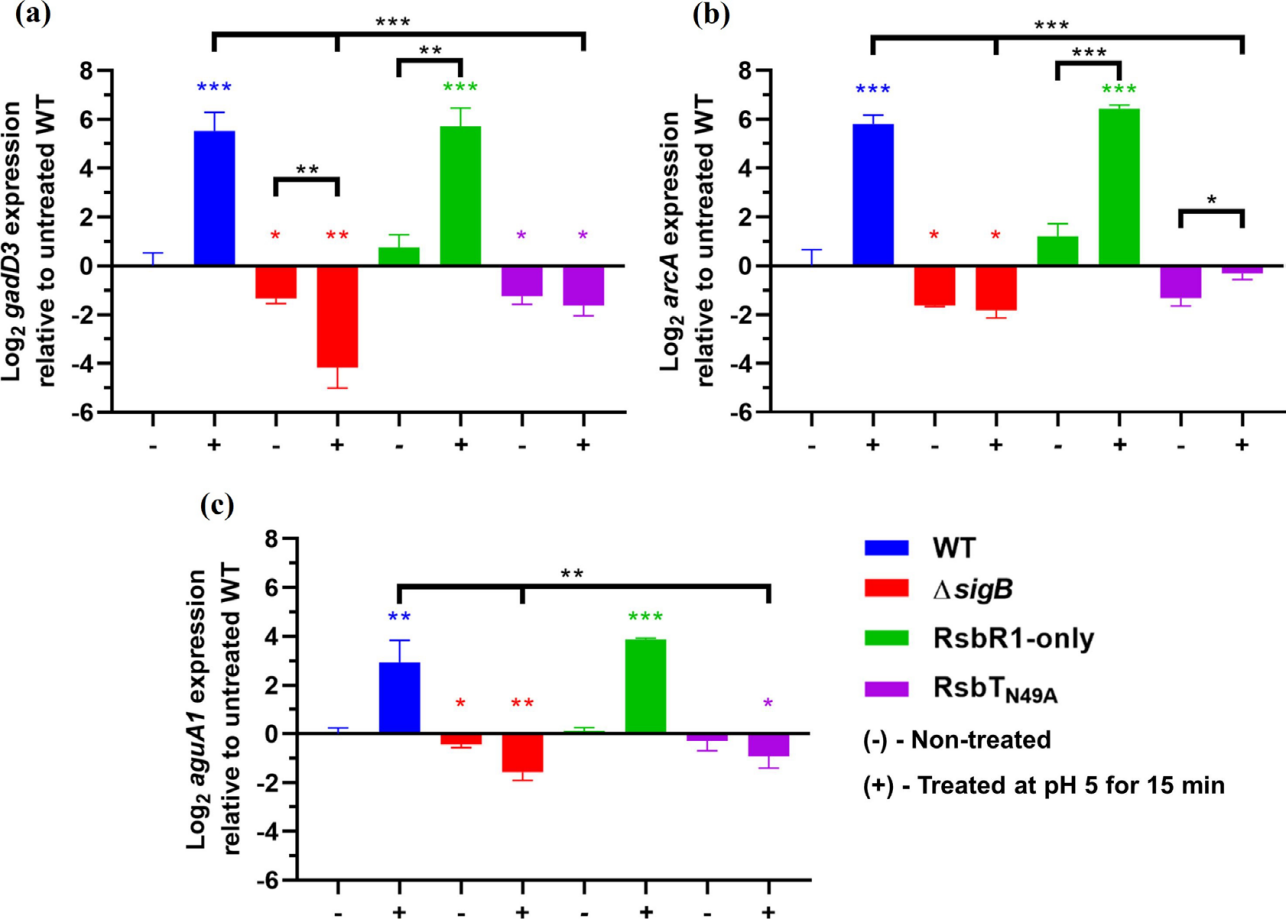
The GAD, ADI and AgDI systems are upregulated by the stressosome and σ^B^ under low pH stress. Mid-log phase cultures (OD_600 nm_ of 0.4) grown at 37°C of *L. monocytogenes* EGD-e wild type, Δ*sigB*, RsbR1-only and RsbT_N49A_ were non-treated (-) and treated (+) at pH 5 for 15 min and the expression of (a) *gadD3*, (b) *arcA* and (c) *aguA1* was measured by RT-qPCR. Three independent biological replicates were made. Transcript levels shown for each gene are expressed relative to the average of those detected in the untreated wild-type strain. Error bars represent sd. Statistical analysis was performed using an unpaired Student *t*-test. Coloured asterisks represent differences relative to the wild-type untreated (-). Black asterisks represent the indicated paired comparisons (*P<0.05; **, *P*<0.01; ***P<0.001).

In this study, we aimed to assess the impact of the stressosome on the regulation of amino acid-based acid tolerance mechanisms in *L. monocytogenes *. First, we analysed transcription of three genes (*gadD3, arcA* and *aguA1*) which are integral parts of the GAD, ADI and AgDI systems, respectively (Fig. 1), in several *L. monocytogenes* mutant strains grown to mid-log phase and then exposed to mild acidic conditions (see Methods). One strain, designated ‘RsbR1-only’, possesses only RsbR1 while the remaining RsbR paralogues were genetically deleted or inactivated [4]. Strain RsbT_N49A_ harbours a single codon substitution in *rsbT* that inactivates its kinase activity [4]. Transcription of *gadD3, arcA* and *aguA1* genes was upregulated (~5.6 log_2_- and ~6.1 log_2_-fold increase for *gadD3* and *arcA*, respectively, and ~3.4 log_2_-fold increase for *aguA1*, *P*<0.05) after the acid shock treatment in both the WT and the RsbR1-only strains (Fig. 2a–c). In the Δ*sigB* and RsbT_N49A_ strains, transcript levels of *gadD3* and *aguA1* were not increased in response to the acid pretreatment in comparison with the treated WT strain (*P*<0.001). However, a small but significant increase was observed for the *arcA* transcript in response to acid in the RsbT_N49A_ strain (*P*<0.05), albeit still well below the level detected in the WT strain. The diminished transcriptional activation of these genes was correlated with the inability to activate σ^B^ via the stressosome, demonstrating that acidic conditions promote the upregulation of *gadD3*, *arcA* and *aguA1* in a stressosome-dependent manner. σ^B^ is crucial for the survival of this bacterium in acidic environments such as the extremely low pH of the human stomach [39–41]. Previous studies found that transcription of *gadD3* and *arcA* is upregulated under mild acidic pH [11, 18]. In addition, Ryan and colleagues identified putative σ^B^ promoters upstream of several genes that comprise the ADI system [18]. However, little was known about the stressosome-mediated activation of σ^B^ and its influence over the transcription of the ADI, AgDI and GAD systems under the same conditions. Our results demonstrate the crucial role of the putative acid sensor RsbR1 and the kinase RsbT, components of the stressosome, in the regulation of these pH homeostatic mechanisms in *L. monocytogenes *. It seems plausible to speculate that this transcriptional upregulation may also increase activity of the GAD, ADI and AgDI systems, but future studies are needed to corroborate these assumptions.

### Transcription of *argR* is unaffected by acid stress in mid-log phase culture

**Fig. 3.**
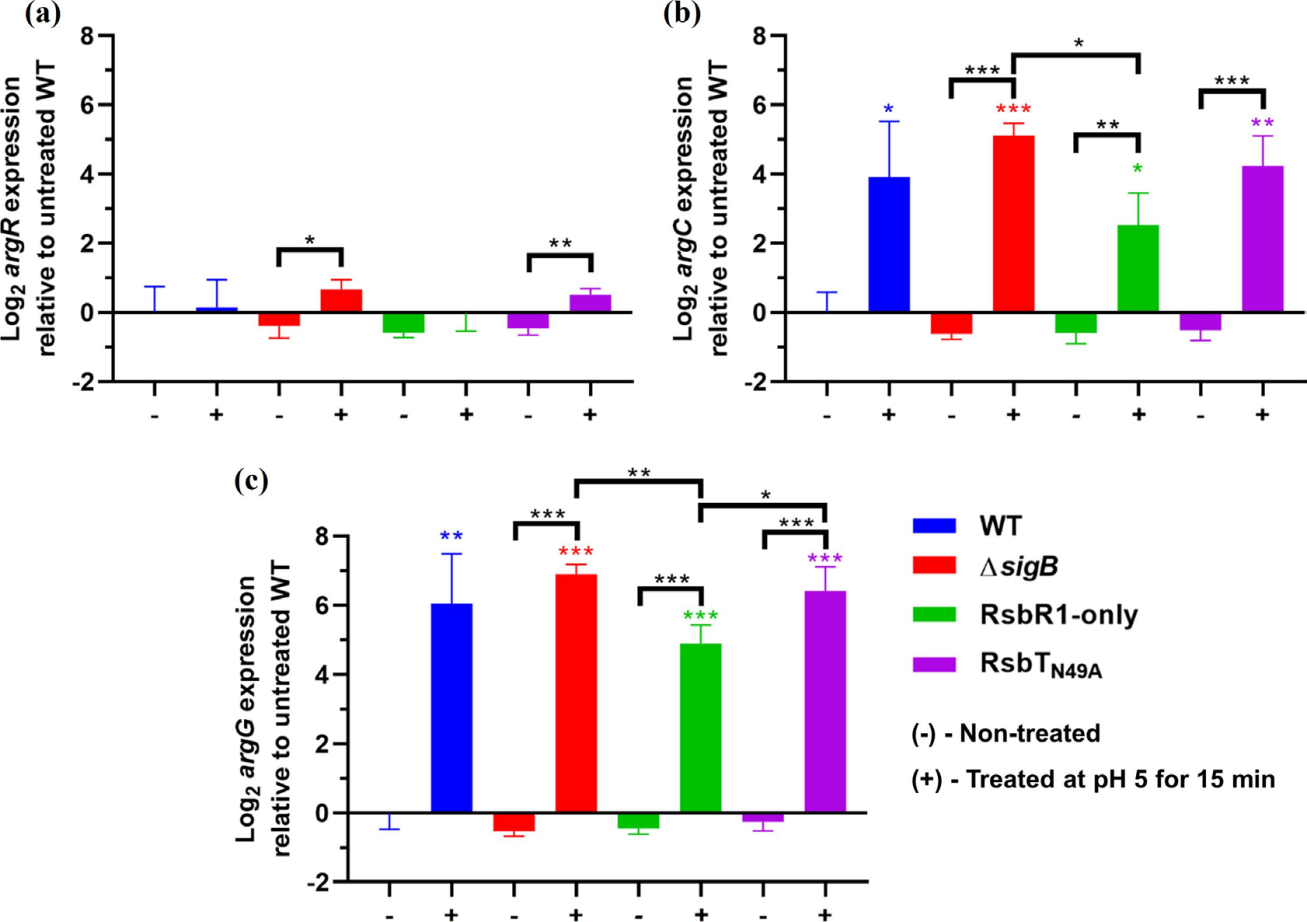
The *argC* and *argG* genes are upregulated independently of the stressosome and σ^B^ under low pH stress. Mid-log phase cultures (OD_600 nm_ of 0.4) grown at 37°C of *L. monocytogenes* EGD-e wild type, Δ*sigB*, RsbR1-only and RsbT_N49A_ were non-treated (-) and treated (+) at pH 5 for 15 min and the expression of (a) *argR*, (b) *argC* and (c) *argG* was measured by RT-qPCR. Three independent biological replicates were made. Transcript levels shown for each gene are expressed relative to the average of those detected in the untreated wild-type strain. Error bars represent sd. Statistical analysis was performed using an unpaired Student *t*-test. Coloured asterisks represent differences relative to the wild-type untreated (-). Black asterisks represent the indicated paired comparisons (*P<0.05; **P<0.01; ***P<0.001).

Similar to σ^B^, ArgR is also required for expression of the ADI system at both neutral and acidic conditions and is strongly upregulated at low pH (5.0–5.5), under anaerobic conditions and in stationary phase grown cells [18, 29]. Furthermore, Ryan and colleagues identified a putative σ^B^ promoter upstream of *argR* [18]. In this study, we aimed to further assess the influence of the stressosome on the transcription of ArgR in mildly acidic conditions. Our results showed no substantial changes in *argR* transcription in mid-log phase cultures treated with acidic shock in either WT or RsbR1-only strains (Fig. 3a). However, a small but significant increase (~0.6 log_2_-fold increase, *P*<0.05) was detected in both Δ*sigB* and RsbT_N49A_ strains (Fig. 3a). Although increased *argR* expression in these two mutant strains was unexpected, it is perhaps not surprising that regulators, other than σ^B^, control the transcription of *argR* during the mid-log phase under stressful conditions and that this control can occur in the absence of σ^B^. Interestingly, anaerobic growth conditions increase the transcription of *argR* [18, 42], which contrasts with the aerobic growth conditions used in our study and conceivably explains the absence of upregulation of the *argR* under acidic conditions. ArgR and σ^B^ may work in concert to upregulate the ADI and AgDI systems, as Ryan and colleagues observed a downregulation of *arcA* in both Δ*sigB* and Δ*argR* strains [18].

### The l-arginine biosynthesis genes *argC* and *argG* are upregulated under mild acid stress independently of the stressosome

In this study, we aimed to evaluate the role of the stressosome under mild acidic conditions on transcription of the first genes of the *arg* biosynthetic operons, *argC* and *argG*. We found that transcription of both genes was highly upregulated (~3.9 log_2_- and ~6.1 log_2_-fold increase, respectively) with acid shock treatment (Fig. 3b, c). The RsbR1-only strain showed slightly lower *argC* and *argG* transcription following acidification (2.5 log_2_- and 5.0 log_2_-fold increase in *argC* and *argG*, respectively) compared with the other strains, and these differences were significant when compared to the Δ*sigB* (*P*<0.05 in *argC* and *P*<0.01 in *argG*) and the RsbT_N49A_ (*P*<0.05 in *argG*) strains. Ryan and colleagues observed increased transcription of *argG* in stationary phase cells treated at pH 5, although an increase in transcription of the negative regulator *argR* was also observed under the same conditions [18]. As ArgR activity is post-translationally regulated by l-arginine, it has been suggested that the repressing action of ArgR is possibly removed following acidification through depletion of the cytoplasmic l-arginine pool [43]. Whether the upregulation of *argC* and *argG* contributes to the acid tolerance in *L. monocytogenes* is still unknown and future studies are needed to assess this question. In *Escherichia coli *, the importation of extracellular arginine contributes to extreme acid tolerance by providing l-arginine for the synthesis of agmatine (reviewed in [44]). In *L. monocytogenes *, the arginine ABC-transporter gene, *arpJ* [45] (encoded by *lmo2250*), was transcriptionally upregulated under acidic conditions [11], suggesting increased import of l-arginine in response to acidification of the medium. Additionally, the acid inducible arginine decarboxylase AdiA, responsible for the conversion of arginine to agmatine, is essential for arginine-dependent acid resistance in *E. coli* [46]. *L. monocytogenes* possesses a putative *adiA* homologue (encoded by *lmo2694*), but its regulation and function have not been characterized in this bacterium. Together, our results show that expression of the *argC* and *argG* genes is upregulated in response to acidification of the medium and independently of the stressosome in mid-log phase grown cells.

Overall, our results show that sensing of pH-related signals by the stressosome is required for upregulation of the GAD, ADI and AgDI systems under mild acidic pH (Fig. 1). We found that for upregulation of *gadD3*, *arcA* and *aguA1* to occur, a functional stressosome with at least RsbR1 is necessary and that the acid-induction of these genes is highly σ^B^-dependent. The increased expression of these systems is probably followed by the increased consumption of protons at the expense of amino acids, culminating in an enhanced tolerance of *L. monocytogenes* to extremely low pH. The arginine biosynthetic genes *argC* and *argG* were upregulated upon acid shock in a stressosome-independent manner, but their role in the adaptability of *L. monocytogenes* to acid remains unknown. Additionally, the role of ArgR in the upregulation of these genes is unclear; whether an alleviation of the repression over *argC* and *argG* due to a decreased level of l-arginine or perhaps due to a decrease in the cytosolic pH remains to be elucidated. Future studies will be required to assess the impact of the stressosome sensing function on the activity of GAD, ADI and AgDI as well as l-arginine biosynthesis, in post-stress environments by assessing the pools of l-arginine in the cells under mild pH stress.
